# Viologen-modified electrodes for protection of hydrogenases from high potential inactivation while performing H_2_ oxidation at low overpotential†
†Electronic supplementary information (ESI) available. See DOI: 10.1039/c8dt00955d


**DOI:** 10.1039/c8dt00955d

**Published:** 2018-05-25

**Authors:** Alaa A. Oughli, Marisela Vélez, James A. Birrell, Wolfgang Schuhmann, Wolfgang Lubitz, Nicolas Plumeré, Olaf Rüdiger

**Affiliations:** a Max-Planck-Institut for Chemical Energy Conversion , Stiftstrasse 34-36 , 45470 Mülheim an der Ruhr , Germany . Email: olaf.ruediger@cec.mpg.de; b Instituto de Catálisis y Petroleoquímica CSIC c/Marie Curie 2 , Cantoblanco 28049 , Madrid , Spain; c Department Analytical Chemistry , Center for Electrochemical Sciences (CES) , Ruhr-Universität Bochum , Universitätsstrasse 150 , 44780 Bochum , Germany; d Center for Electrochemical Sciences—Molecular Nanostructures , Ruhr-Universität Bochum , Universitätsstrasse 150 , 44780 Bochum , Germany . Email: nicolas.plumere@rub.de

## Abstract

In this work we present a viologen-modified electrode providing protection for hydrogenases against high potential inactivation. Hydrogenases, including O_2_-tolerant classes, suffer from reversible inactivation upon applying high potentials, which limits their use in biofuel cells to certain conditions. Our previously reported protection strategy based on the integration of hydrogenase into redox matrices enabled the use of these biocatalysts in biofuel cells even under anode limiting conditions. However, mediated catalysis required application of an overpotential to drive the reaction, and this translates into a power loss in a biofuel cell. In the present work, the enzyme is adsorbed on top of a covalently-attached viologen layer which leads to mixed, direct and mediated, electron transfer processes; at low overpotentials, the direct electron transfer process generates a catalytic current, while the mediated electron transfer through the viologens at higher potentials generates a redox buffer that prevents oxidative inactivation of the enzyme. Consequently, the enzyme starts the catalysis at no overpotential with viologen self-activated protection at high potentials.

## Abstract 2

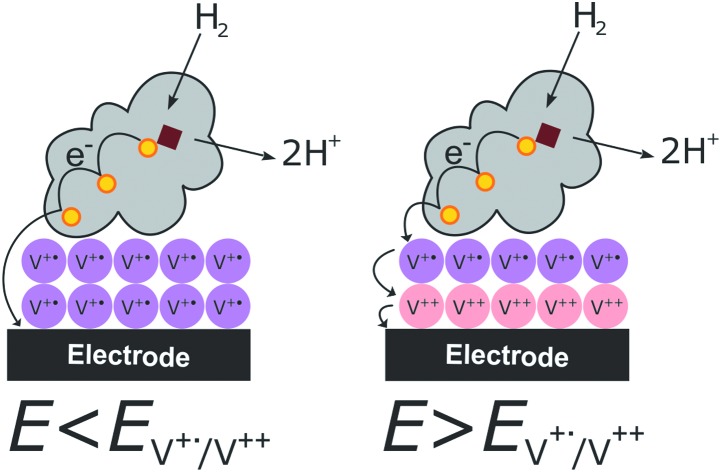
In this work we present a viologen-modified electrode providing protection for hydrogenases against high potential inactivation.

## Introduction

Interest in hydrogen as an energy carrier has increased rapidly in the search for a clean and sustainable energy economy.^1^–^3^ Platinum is, to date, the most efficient catalyst used in hydrogen-based technological devices for energy conversion. However, the scarcity of Pt and other noble metals prohibits their implementation on a global scale. Hydrogenases are nature's catalysts for both hydrogen oxidation and production.^4^ Despite their high rates of H_2_ oxidation activity that are comparable to that of Pt,^5^ hydrogenases suffer from various oxidative inactivation pathways that limit their use in technological devices.^6^ The majority of hydrogenases (except an O_2_ tolerant class) are rapidly inactivated by trace amounts of oxygen.^7^,^8^ Hydrogenases are also inactivated upon exposure to high potentials. This inactivation pathway also affects O_2_-tolerant hydrogenases^4^,^9^–^12^ In the case of [FeFe] hydrogenases, high potential inactivation was recently shown to be strongly influenced by chloride or bromide anions in the electrolyte.^13^

Mediated electron transfer from hydrogenases to electrodes through redox active polymeric structures was previously described in several reports.^14^–^16^ We have proposed the use of a viologen-based redox hydrogel for wiring hydrogenases to an electrode surface.^17^–^19^ The redox hydrogel physically entraps the hydrogenase in the polymer matrix where the viologen moieties serve as electron relays between the enzyme and the electrode surface. Moreover, we designed the redox hydrogel films to protect the enzyme from both inactivation pathways, namely high potential inactivation and oxygen damage. A major limitation of the hydrogel is that the onset potential of hydrogen oxidation activity is imposed by the redox potential of the viologen. This results in an energy loss (*e.g.* at pH 7 *E*_2H^+^/H_2__ = –413 mV, *E*_V^+^/V^2+^_ = –300 mV, overpotential = 113 mV) and consequently a power loss when the electrode is employed in a fuel cell.

In this work, we report an alternative method for immobilizing hydrogenases on an electrode surface, while retaining the protection from high potential inactivation. A viologen derivative with an aromatic amine functionality is covalently attached to an electrode surface. Afterwards, the hydrogenase is immobilized on the viologen layer preferentially with the correct orientation for efficient electron transfer due to electrostatic interactions similar to what has been described for other hydrogenases.^20^–^23^ Mediated electron transfer through the viologen layer provides a Nernst potential buffer, preventing oxidative potentials being applied to the hydrogenase. Moreover, in this configuration, the hydrogenase can also engage in direct electron transfer with the electrode surface, unlike in the redox hydrogel. This enables catalytic H_2_ oxidation activity to start at the thermodynamic equilibrium potential for hydrogen.

## Results and discussion

### Synthesis of the viologen derivative

**Scheme 1:**
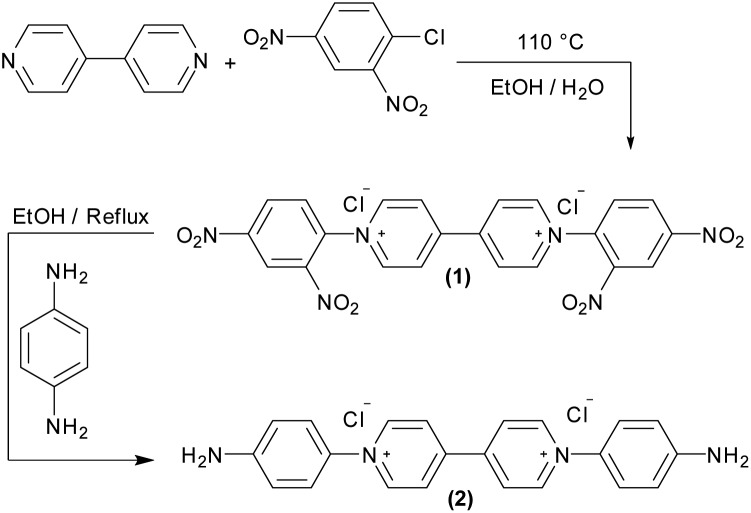
Synthesis of the viologen derivative 1,1′-bis(4-aminophenyl)-[4,4′-bipyridine]-1,1′-diium chloride (**2**).

Viologens were chosen because of their efficient electron transfer with hydrogenases, their stability and high degree of reversible redox behavior.^17^,^19^,^24^,^25^ The viologen derivative used in this study was designed to have functionalities that enable its covalent immobilization on an electrode surface, as well as providing attractive electrostatic interactions with hydrogenases. Aromatic amines allow the formation of diazonium salts that can be electrochemically reduced and attached to an electrode surface. Hence, a symmetric viologen derivative (**2**) functionalized with an aromatic amine was synthesized in a similar manner as previously reported^26^,^27^ (Scheme 1).

### Electrode modification and electrochemical characterization

**Fig. 1:**
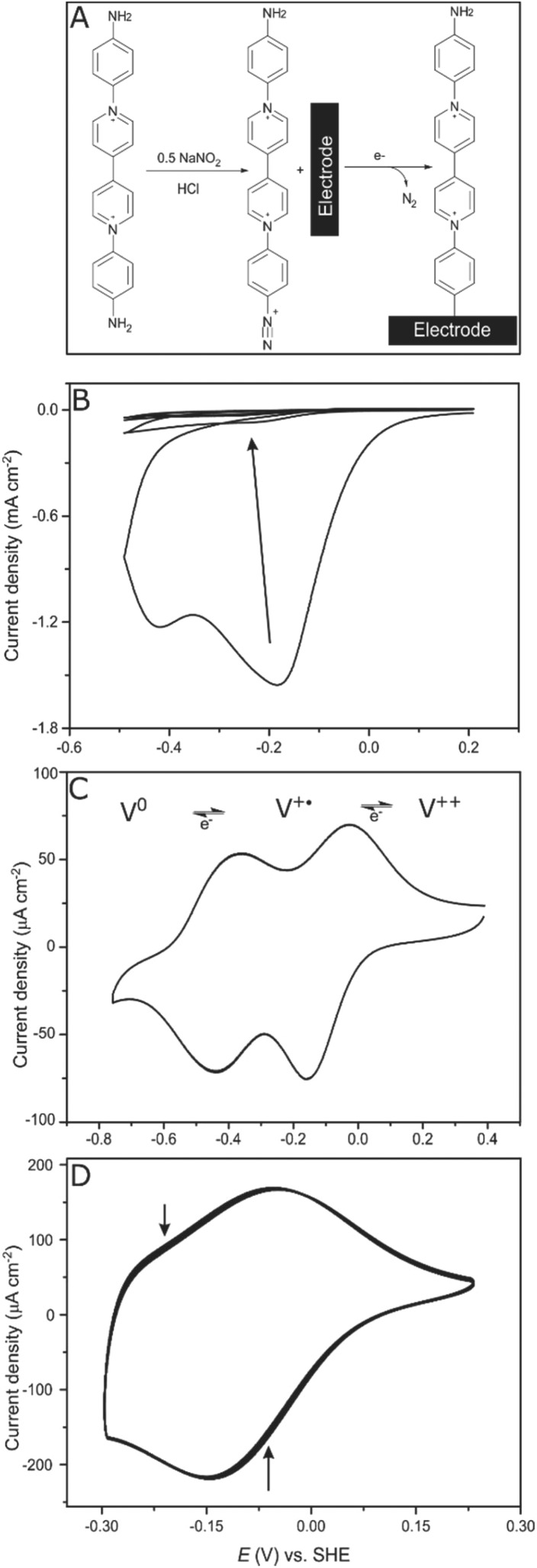
Electrode modification. (A) A scheme representing the diazonium salt formation followed by electrochemical reduction and attachment of the viologen derivative on the electrode. (B) Electrochemical reduction of the *in situ* generated diazonium salt on a glassy carbon electrode (GCE). The electrolyte contains 0.024 mmol of the viologen derivative (**2**) and 0.014 mmol NaNO_2_ dissolved in 2 mL of HCl 0.5 N. Conditions: 25 °C, 0.1 V s^–1^, 4 cycles. (C) A CV of a modified GC electrode in aqueous buffer mix pH 7, 25 °C, 50 mV s^–1^ and under N_2_. (D) Viologen stability on the electrode surface. 100 CVs of a gold electrode covalently modified with viologen. Arrows on the CVs indicate the trend in current upon repeated sweeps. Conditions: 25 °C, aqueous buffer pH 7, N_2_, 0.1 V s^–1^.

Electrodes (glassy carbon (GCE) as well as gold electrodes) were covalently modified by electrochemical reduction of the *in situ* generated diazonium salt of the viologen derivative (**2**) (Fig. 1A). Less than half an equivalent of NaNO_2_ was added to ensure that only one side of the symmetric viologen is transformed to a diazonium salt while the amine functionality on the other side stays unreacted.

The freshly generated diazo group can be easily electrochemically reduced, N_2_ is released and the generated aryl radical rapidly reacts with the electrode surface forming a strong C–C bond.^28^,^29^ Controlling the number of scans and the potential range during cyclic voltammetry enables the controlled growth of multilayers of the viologen derivative; *in situ* formed aryl radicals can attack the viologen moieties that are already attached to the surface, which results in chains of viologens forming a thick layer. Four cyclic voltammetry scans were recorded in the range (+0.2 to –0.5 V, *vs.* SHE) to ensure a full coverage of the electrode surface (Fig. 1B).

After modification, the electrode was ultra-sonicated in water to remove any non-covalently attached viologen from the surface; a cyclic voltammogram of this electrode at pH 7 shows two redox signals responsible for the first (V^++^/V^+^˙) and the second (V^+^˙/V^0^) redox transitions of the viologen at –110 and –420 mV *vs.* SHE, respectively, which confirms that the electrode is modified with the viologen moiety (Fig. 1C). The redox signal intensity of the viologen multilayer remains unchanged for 100 cycles with a scan rate of 100 mV s^–1^ indicating the stability of the viologen moieties on the electrode surface when scanning in the range of the first redox signal (*E*_V^++^/V^+˙^_ = –110 mV *vs.* SHE) (Fig. 1D). On the other hand, when scanning to more negative potentials, covering the second reduction peak, the signal intensity decayed over the course of the experiment (ESI Fig. S2†). This lack of stability for the viologen system on the second reduction process has already been observed in viologen polymers.^30^

By integrating the oxidation signal of the V^++^/V^+^˙ pair, the coverage (*Γ*) of the gold electrode was calculated to be 3.9 × 10^–9^ mol cm^–2^; a similar value was obtained for a glassy carbon electrode (GCE), which is in accordance with *Γ* of a similar reported system.^31^ This value corresponds to a coverage of ≈25 molecule per nm^2^. This can only be explained by the existence of multilayers of viologen in contrast to the previously reported viologen self-assembled monolayer on gold,^32^–^34^ covalently attached viologen monolayer on GCE^35^ or even monolayers of similar-sized molecules such as quinone derivatives^36^ where a coverage of about 2 molecules per nm^2^ was reported.

Cyclic voltammograms of the V^++^/V^+^˙ couple were recorded at different scan rates (5 to 250 mV s^–1^): both cathodic and anodic peak currents (*i*_p_) increased with the scan rate (ESI Fig. S1-A†). The linearity of the peak current *vs.* scan rate is characteristic of a surface confined species where all viologen moieties on the electrode surface are contributing to the redox process on the time scale of the experiment and no diffusion-controlled processes are taking place (ESI Fig. S1-B†).

**Fig. 2:**
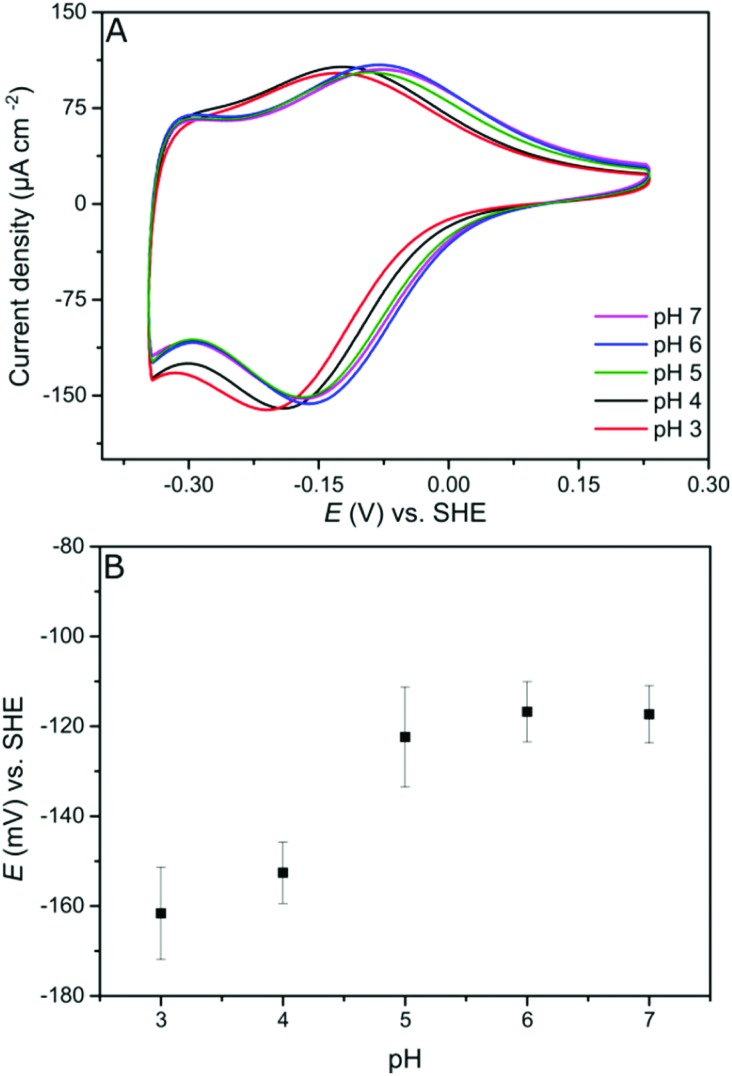
Redox potential at different pH values. (A) CVs of a viologen-modified GCE at different pH values. (B) Viologen redox potential *vs.* pH (*n* = 3). Conditions: aqueous mix buffer, 100 mV s^–1^, 25 °C, N_2_.

Since the viologen moieties have an amine functionality, their redox potential is mildly influenced by pH changes. In the range between pH 5 and pH 7 the potential shift is very small. However, lowering the pH to 4 results in a larger shift to more negative redox potential (Fig. 2). The negative shift was unexpected but may be a consequence of the fact that unprotonated amine groups conjugate with the aromatic π-system of the viologen moiety.^37^ Protonation breaks this conjugation and so electron delocalization into the amine groups is restricted, resulting in a destabilization of the radical cation state,^38^ and hence a lower potential.^39^

### Atomic force microscopy measurements

**Fig. 3:**
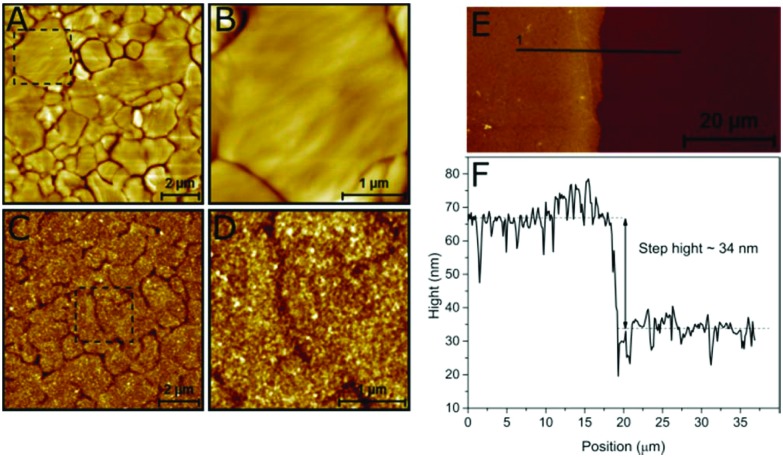
AFM topographic images of a bare (A, B) and a viologen-modified (C, D) Au-coated substrate surface with different scanning sizes. AFM topographic image of a viologen-modified Au-coated substrate surface 70 × 70 μm^2^ showing the edge between the modified and bare gold (E) and calculation of the step height from the line profile (F).

The structure of the viologen multilayer was examined by atomic force microscopy (AFM). A gold-coated substrate was prepared as described in the Experimental section to produce flat Au (111) terraces with different sizes that could be identified by AFM (Fig. 3A and B); then the gold surface was modified electrochemically with the viologen derivative. The viologen-modified gold surface has an appearance that is very different from the bare gold. The borders of the terraces can still be distinguished, which indicates that the layer is compact and attached closely to the Au surface; however, the surface is rougher and has a more detailed structure resulting from the branching of the viologen chains (Fig. 3C and D).

During surface modification, the Au-coated substrate was not fully immersed in the film-formation solution leaving a region of unmodified surface. The thickness of the viologen layer was measured by scanning the interface between the modified and bare gold. The step height was calculated to be around 34 nm using the line profile of the boundary (Fig. 3E and F), which represents the thickness of the viologen layer. The thicker edge probably reflects a slight increase in deposited material at the wetting front of the buffer on the Au-coated substrate. Previously reported self-assembled monolayers of viologen derivatives had a thickness of ≈7 Å.^35^ This indicates that we have created multilayers of viologen, supporting the electrochemical-based calculation of the surface coverage discussed above.

ESI Fig. S3† shows a 3D comparison between a bare and a viologen-modified Au-coated substrate with and without protein adsorbed. Before modification, a surface with a roughness of less than 1 nm was obtained. Modification with viologen increased the surface roughness up to 15 nm. The profile after addition of *Desulfovibrio desulfuricans* [FeFe] hydrogenase (*Dd*HydAB) is flatter, indicating that the protein inserts and fills the holes of the viologen layer.

### Hydrogenase in direct electron transfer configuration

**Fig. 4:**
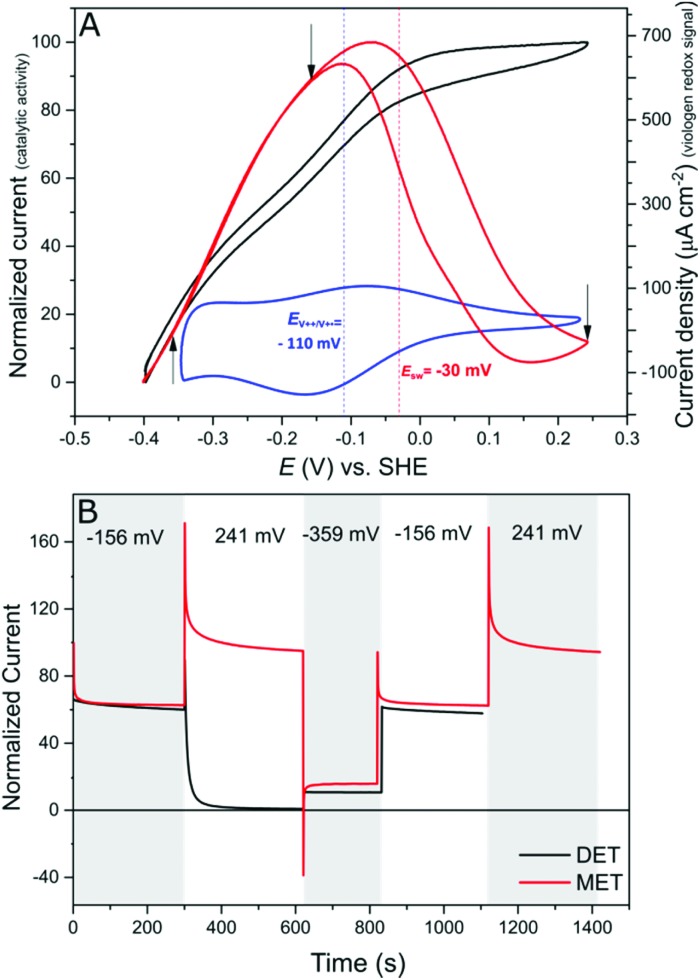
High potential deactivation/protection. (A) Comparison of cyclic voltammograms of a PGE modified with *Dd*HydAB in a DET configuration (red) and of a *Dd*HydAB adsorbed on a viologen-modified GCE (black). The value of the switch potential *E*_sw_ is indicated with a dashed red line. The dashed blue line shows the mid potential for the immobilized viologen, determined from the CV shown in blue. Conditions: 1000 rpm, aqueous buffer mix pH 7, 25 °C, 5 mV s^–1^ and 1 bar of H_2_. (B) Chronoamperometry experiments of *Dd*HydAB on a graphite electrode (black) and on the viologen-modified electrode (red) with application of different potentials (indicated by arrows on A). Conditions: 25 °C, aqueous buffer mix pH 7, 1000 rpm, 100% H_2_.

Protein film electrochemistry (PFE) is the method of choice to study the electrocatalytic activity of hydrogenases. In PFE the enzyme is adsorbed on an electrode surface and the electric currents arising from catalytic activity are recorded as a function of applied potential.^40^*Dd*HydAB was adsorbed on a bare pyrolytic graphite electrode (PGE) as described in the Experimental section. In this configuration, the enzyme exhibits both hydrogen oxidation and production as a result of direct electron transfer between the enzyme and the electrode surface, as was described previously.^41^ Consequently, the enzyme experiences the potentials applied at the electrode surface. Hydrogenases are inactivated at high potentials forming oxidized states;^9^,^10^,^42^ however, the enzyme can be reactivated by applying reductive potentials. This behavior can be seen in Fig. 4A (red trace) where the catalytic H_2_ oxidation current decreases at high potentials. The potential at which the enzyme reactivates is referred to as the switch potential (*E*_sw_). This potential can be defined as the potential of the maximum slope of the reversed scan and is calculated from a derivative plot (ESI Fig. S4†). We determined an *E*_sw_ for *Dd*HydAB of –30 mV *vs.* SHE at a scan rate of 5 mV s^–1^ and pH 7, similar to what was reported before for this enzyme (Fig. 4A, dotted red line).^43^

### Hydrogenase on the viologen-modified electrode

**Fig. 5:**
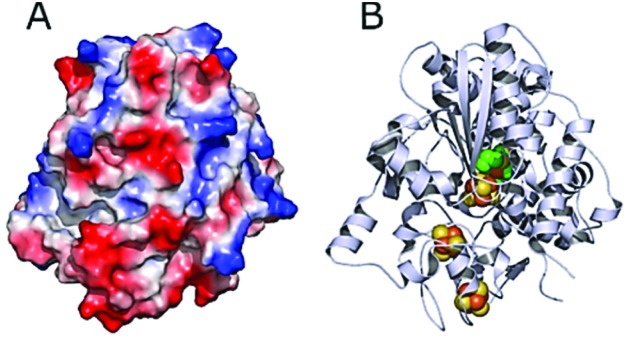
Structure of *Dd*HydAB (PDB ; 1HFE).^44^ (A) Surface vacuum electrostatic potential distribution of *Dd*HydAB showing negative regions (red) and positive regions (blue) (calculated with PyMol). (B) 3D structure of *Dd*HydAB showing the deeply buried active site (green/yellow), the iron-sulfur clusters (orange/yellow) and the protein shell (gray).

*Dd*HydAB is negatively charged on the enzyme surface close to the distal Fe–S cluster of the electron transfer relay (Fig. 5).^44^ The electrostatic interaction between the highly positively charged viologen-modified surface and the enzyme's electron transfer “gate” allows immobilization of the enzyme with the correct orientation for efficient electron transfer.

**Fig. 6:**
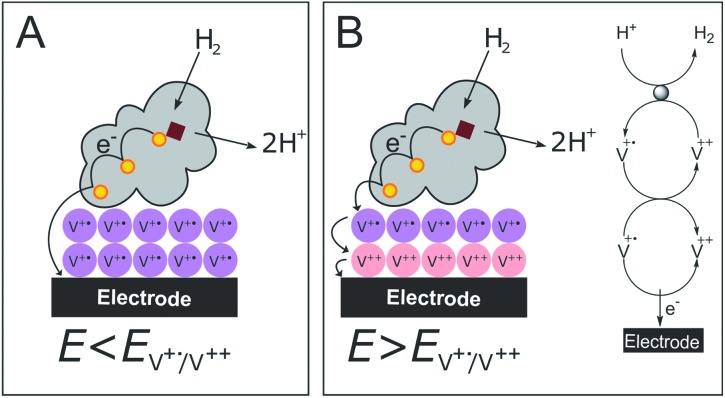
Cartoon representing the viologen-modified electrode at electrode potentials lower (A) and higher (B) than *E*_V^++^/V^+˙^_.

In contrast to the DET configuration (Fig. 4A, red trace), when *Dd*HydAB is immobilized on the viologen-modified surface, the catalytic H_2_ oxidation current does not decrease at potentials higher than *E*_sw_ and a plateau is reached at +30 mV and remains stable for potentials as high as +240 mV *vs.* SHE (Fig. 4A, black trace). At electrode potentials lower than *E*_V^++^/V^+˙^_, the viologen is in its reduced state (V^+^˙) and there is no driving force for it to accept electrons from the enzyme. Nevertheless, the catalytic activity onset potential for *Dd*HydAB on the viologen-modified surface starts at –413 mV *vs.* SHE at pH 7, which is the thermodynamic potential for the H_2_ couple and identical to the onset potential of the DET configuration, since these enzymes operate at zero overpotential. This indicates that electrons are still able to tunnel directly from the enzyme to the electrode surface in spite of the presence of the viologen layer (Fig. 6A). The DET can be explained by the previously discussed AFM images where the enzyme filled the deep valleys in the viologen-modified surface terrains in a range where direct tunnelling of electrons can take place. Similar mixed MET/DET behavior was reported earlier for a hydrogenase adsorbed on an electropolymerized toluidine blue layer. However, protection from high potential inactivation was not investigated in that work.^45^

In addition to the well-described reversible high potential inactivation of hydrogenases^9^–^12^ we found an additional irreversible inactivation process taking place at extremely high potentials (*E* > +500 mV *vs.* SHE). *Dd*HydAB in DET configuration is rapidly deactivated after applying potentials as high as 600 mV *vs.* SHE and almost no activity is recovered upon decreasing the potential. In contrast, *Dd*HydAB on a viologen-modified electrode deactivates much slower, under the same conditions, and more than 50% of its activity can be recovered after the same treatment (ESI Fig. S5†), demonstrating the effectiveness of the protection mechanism.

**Fig. 7:**
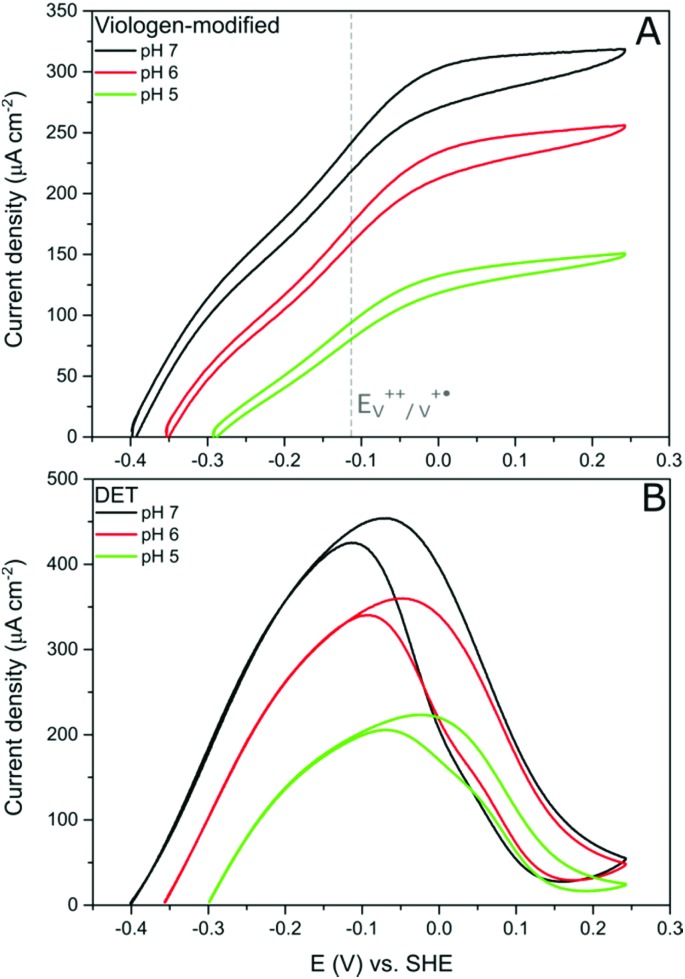
Comparison of cyclic voltammograms of *Dd*HydAB immobilized on viologen-modified GCE (A) and in a DET system on a PGE (B) at three different pH values in an aqueous buffer. Conditions: 5 mV s^–1^, 25 °C, 1000 rpm, 100% H_2_.

Further evidence of the DET process at low potentials can be obtained from observing how the overpotential for the catalytic activity changes with altering the buffer pH value. It was shown earlier that the viologen redox potential remains essentially constant in the range pH 5–pH 7 (Fig. 2A). Consequently, if the viologen layer is responsible for electron transfer, then the catalytic activity onset potential should *not* shift with the pH in this range. In fact, Fig. 7 shows this not to be true: the onset potential for *Dd*HydAB catalytic activity in the viologen-modified electrode system shifts ≈60 mV to more positive values when decreasing the pH value by one unit (Fig. 7A). Identical behavior with the same onset potential for the corresponding pH is observed for the DET configuration (Fig. 7B), which supports the interpretation that a DET process is taking place in the viologen-modified electrode at electrode potentials lower than *E*_V^++^/V^+˙^_.

## Conclusion

We have shown that electrodes modified covalently with a viologen derivative do not exhibit any overpotential to drive catalytic H_2_ oxidation by hydrogenases, while protecting them from high potential deactivation. These advantages are a result of the interplay of the surface viologen molecules and the enzyme, which results in a mixed system of direct electron transfer at low potentials and mediated electron transfer at higher potentials. Using this method of protection eliminates the requirement of cathode-limiting conditions when employing hydrogenases in biofuel cells^46^ and allows working under anode-limiting conditions.

## Experimental section

### Synthesis of the viologen derivative 1,1′-bis(4-aminophenyl)-[4,4′-bipyridine]-1,1′-diium chloride (**2**)

All materials for viologen derivative synthesis were bought from Sigma-Aldrich and used with no further purification. 4,4′-Bipyridine (1.56 g, 10 mmol) and 2,4-dinitro-chlorobenzene (5.66 g, 28 mmol) were melted together in a round bottomed flask without solvent at 110 °C for 10 min, then 9 ml of ethanol/water mixture (1 : 1) was added to the melt and the reaction mixture was refluxed for 15 h at 110 °C. After complete removal of the solvent, the dark green crude product was recrystallized from ethanol by adding few drops of water to get the intermediate compound 1,1′-bis(2,4-dinitrophenyl)-[4,4′-bipyridine]-1,1′-diium chloride (**1**) as a greenish grey powder. **1** (0.5 g, 0.89 mmol) was dissolved in 250 mL of ethanol in a round bottomed flask with a magnetic stirrer. To this solution *p*-phenylenediamine (281 mg, 2.6 mmol) was added and the mixture was refluxed under argon for 6 h. After letting the mixture cool down, the solvent was removed by a rotary evaporator and the black residue was washed extensively with THF, followed by acetone, and then dried under high vacuum to give (**2**) as a black powder (366 mg, 0.85 mmol, 96% yield). ^1^H NMR (500 MHz, D_2_O) *δ* 9.16 (d, *J* = 6.3 Hz, 4H), 8.57 (d, *J* = 6.3 Hz, 4H), 7.48 (d, *J* = 8.3 Hz, 4H), 6.93 (d, *J* = 8.3 Hz, 4H).

### Enzyme purification

*Desulfovibrio desulfuricans* [FeFe] hydrogenase (*Dd*HydAB) was over-produced in *Escherichia coli*, purified, artificially maturated and characterized as described previously.^41^

### Electrode modification

Prior to electrode modification, glassy carbon (GC) and gold electrodes were polished with alumina slurry (1 and 0.05 μm) then washed with ultrapure water and sonicated for 5 min. Additionally, gold electrodes were electrochemically cleaned in 0.5 M H_2_SO_4_ by scanning from 0 to 1.6 V at 50 mV s^–1^ for 50 cycles. Gold as well as GC electrodes were modified with the viologen derivative by reduction of the *in situ* generated diazonium salt. The electrochemical cell was placed in an ice-bath and charged with **2** (10 mg, 0.024 mmol) and dissolved in 2 mL of HCl (0.5 M). This solution was bubbled with argon, then NaNO_2_ was added (1 mg, 0.014 mmol) and the mixture was left to react for 5 min prior to the electrochemical immobilization (ESI Fig. S6† for the NMR of the diazonium salt formation reaction). The modification was accomplished by five consecutive cyclic voltammetry scans from 0 to –0.7 V *vs.* Ag/AgCl 3 M. After modification, the electrodes were washed with ultrapure water then sonicated in water.

### Enzyme immobilization

The viologen-modified electrode was covered with a droplet of MES buffer (0.1 M, pH 6) for 5 min. Afterwards, 5 μL of 50 μM *Dd*HydAB in 10 mM MES buffer (pH 6.5) were placed on top of the electrode for 1 h. The electrode was then rinsed with buffer (pH 7) and placed in the electrochemical cell for measurements. For DET measurements, the hydrogenase was adsorbed on PGE polished with sandpaper; 5 μL of 50 μM *Dd*HydAB in 10 mM MES buffer (pH 6.5) were placed on the electrode surface and left for 1 hour, the electrode was then rinsed with water and placed in the electrochemical cell for measurements.

### Electrochemical measurements

All electrochemical measurements involving hydrogenase were performed in a glovebox filled with N_2_ (MBraun) and were recorded on a VersaStat 4-400 potentiostat (Princeton Applied Research). A three-electrode gas tight electrochemical cell was used with a saturated calomel electrode (SCE) located in the side arm as the reference electrode and a Pt wire as the counter electrode. A gas flow controller was used to control O_2_, N_2_ and H_2_ (Air Liquide S.A.) flow. As an electrolyte, buffer mix (MES, HEPES, TAPS, CHES, NaOAc, 15 mM each) and NaCl (0.1 M) was used. Electrochemical electrode modification was performed outside the glove box and a three-electrode cell was used with Ag/AgCl (3 M) as a reference electrode and Pt wire as a counter electrode.

### Atomic force microscopy

An Agilent Technologies 5500 (Santa Clara, CA) microscope was used for AFM imaging with Olympus rectangular silicon nitride cantilevers. Images were taken in contact mode and processed using SPIP™ software (V 6.6.5).

### Gold substrate preparation for AFM measurements

Au-coated substrates (11 × 11 mm^2^) (Arrandee metal GmbH+Co.) were first soaked in piranha solution (three parts H_2_SO_4_ 98% and one part H_2_O_2_ 30%) and rinsed with water. The substrates were then annealed in a methane flame until glowing dark red, then they were removed from the flame and left to cool down for 30 seconds; this operation was repeated three times. With this procedure, we could obtain a surface containing Au (111) terraces of a radius of 100 nm to a few μm suitable for AFM measurements. The gold substrate was then modified electrochemically following the same procedure for electrodes as described above.

## Conflicts of interest

There are no conflicts to declare.

## Supplementary Material

Supplementary information
