# Supplementary material for: Viologen-modified electrodes for protection of hydrogenases from high potential inactivation while performing H2 oxidation at low overpotential
Source: Dalton Trans. 2018 May 25;47(31):10685–91. doi: 10.1039/c8dt00955d (PMC6083823; doi:10.1039/c8dt00955d)
Supplement: Supplementary file 1 [file DT-047-C8DT00955D-s001.pdf]

Electronic Supplementary Material (ESI) for Dalton Transactions.
This journal is © The Royal Society of Chemistry 2018

Supplementary Information for: 'Viologen-modified Electrodes for Protection of Hydrogenases from High Potential Inactivation while Performing H_2 Oxidation at Low Overpotential' by Alaa A. Oughli, Marisela Vélez, James Birrell, Wolfgang Schuhmann, Wolfgang Lubitz, Nicolas Plumeré and Olaf Rüdiger

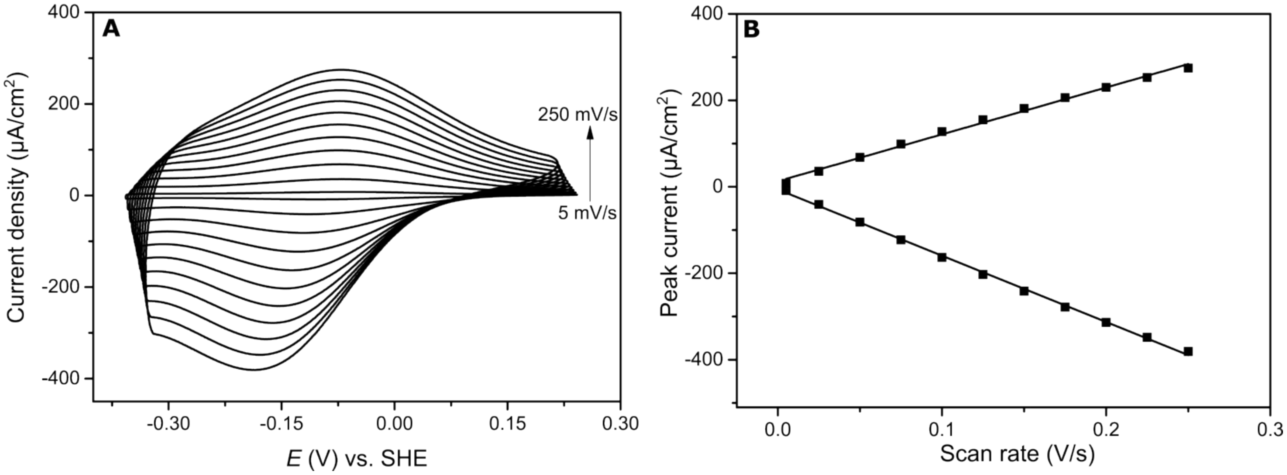

Figure S1. Electrode characterization. A) CVs of a gold electrode modified covalently with viologen with scan rates of 5, 25, 50, 75, 100, 150, 175, 200 and 250 mV s^{-1}. B) Anodic and cathodic peak currents versus scan rate showing a linear response. Conditions: aqueous buffer pH 7, 25°C, N_2.

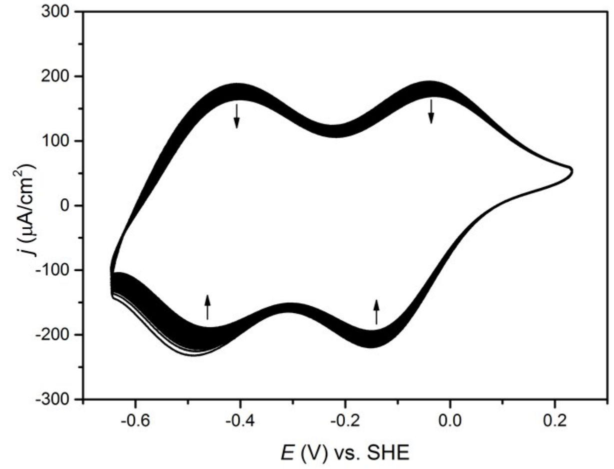

Figure S2. Viologen stability of the film at negative potentials. 100 consecutive CVs of a Ag electrode modified with a viologen film. Conditions: 25ºC, aqueous buffer pH 7, N_2, 0.1 V s^{-1}.

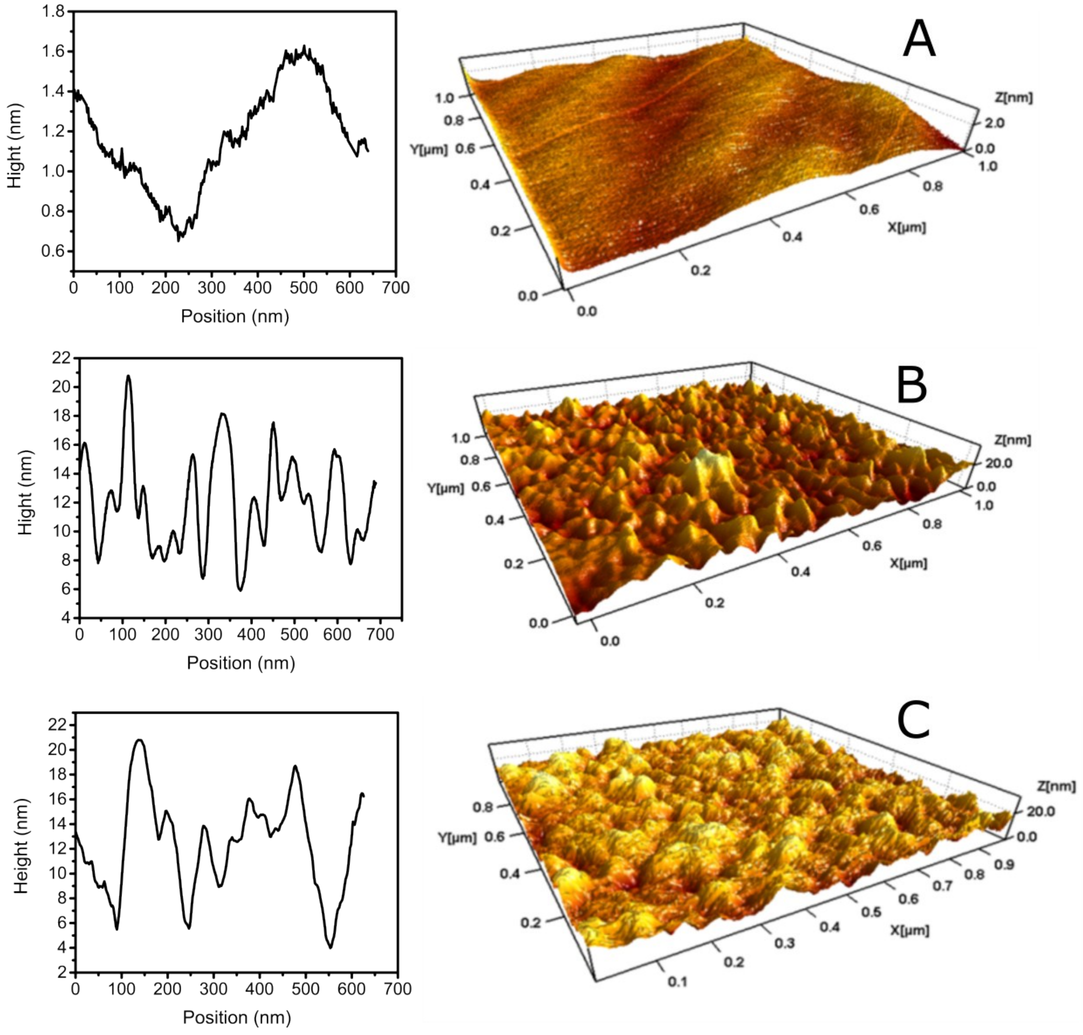

Figure S3. AFM topographic images, in 3D representation of a bare (A), viologen modified (B) and protein modified (C) 1x1 μm^2 area of a Au-coated substrate. A line profile is shown on the left.

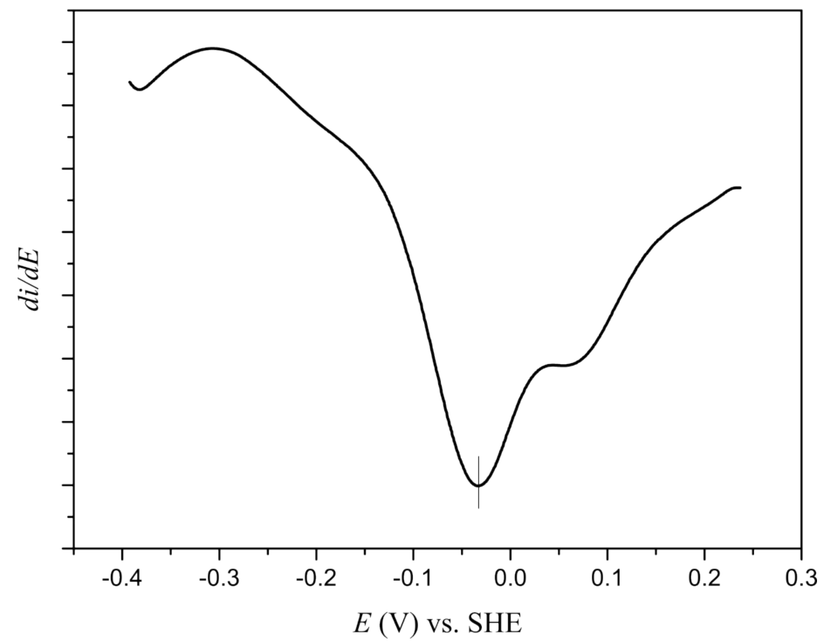

Figure S4. Determination of the E_{sw} using the derivative of the reversed scan of the CV of *Dd*HydAB activity. The second more positive peak is attributed to another inactivation process, which is still currently under investigation.

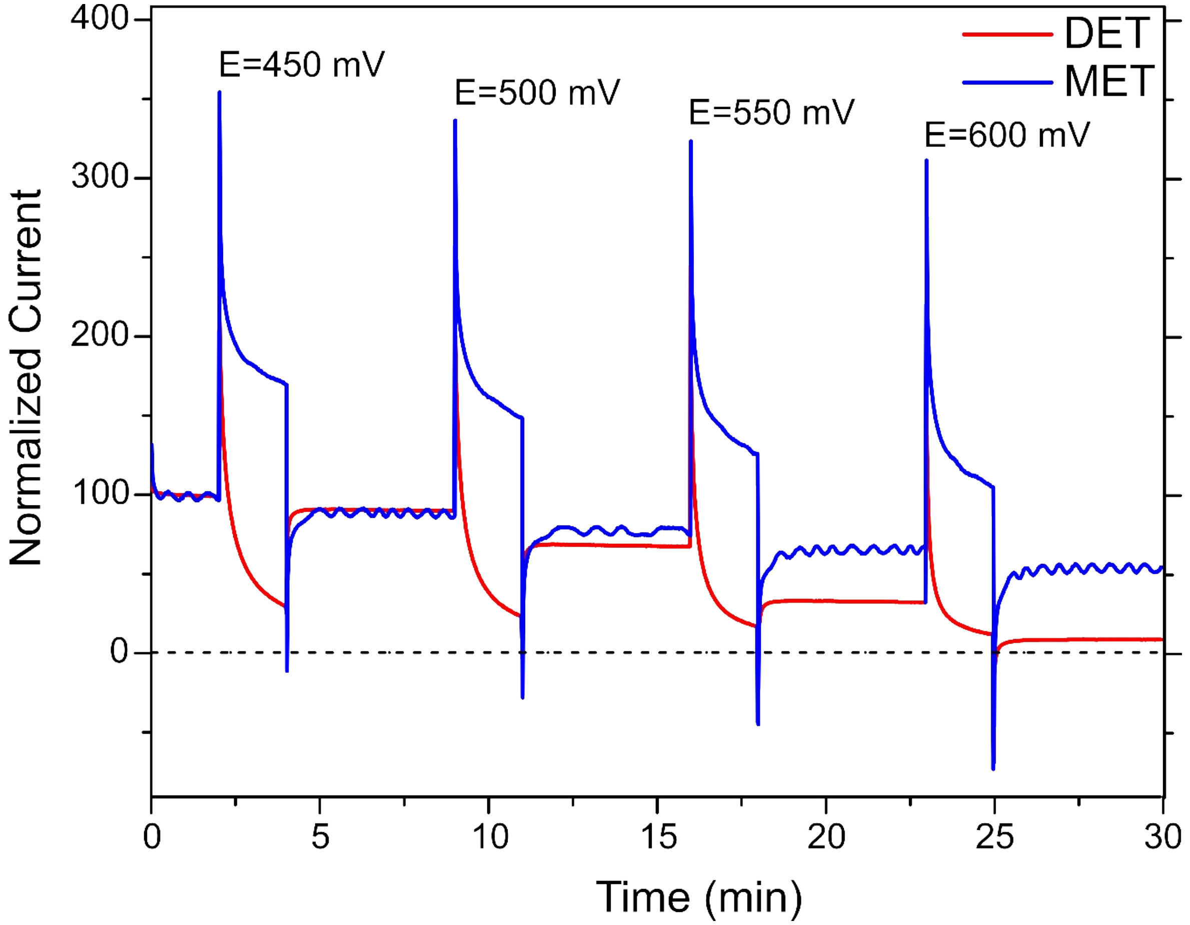

Figure S5. Chronoamperometry experiment of *Dd*HydAB in DET mode on PGE (red) and on a viologen-modified GCE (blue). Potentials of 450, 500, 550, 600 mV vs. SHE were applied for 2 min each and a potential of -170 mV vs. SHE was applied for 5 min after each high potential to reactivate the enzyme. Conditions: 25°C, aqueous buffer pH 7, 1000 rpm, 100% H_2.

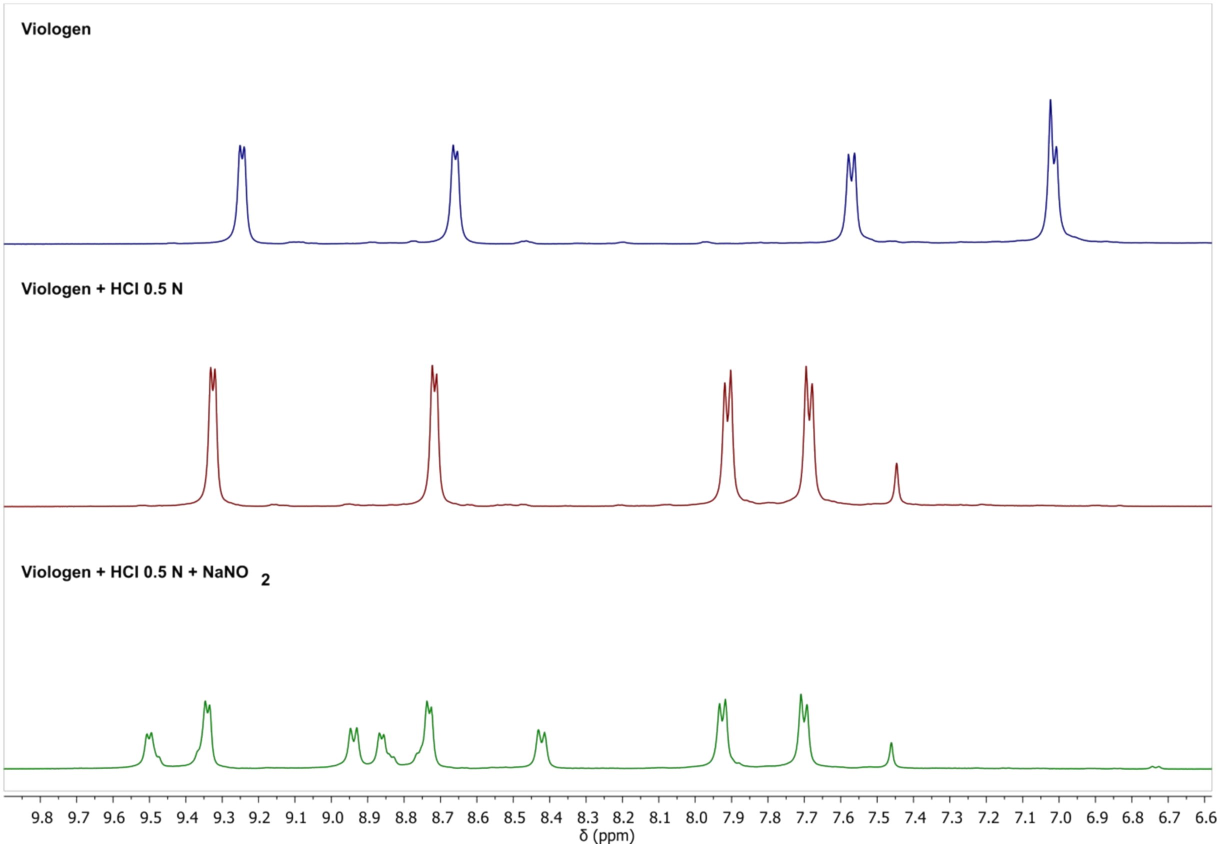

Figure S6. ^{1}H NMR spectra of the viologen derivative **(2)** in D_2O (blue), in HCl 0.5 N solution in D_2O (red) and with addition of $NaNO_2$ (green). The new peaks after $NaNO_2$ addition appear as a result of forming the diazonium salt of the viologen.
